# Googling the Lifetime Risk of Stroke Around the World

**DOI:** 10.3389/fneur.2020.00729

**Published:** 2020-07-31

**Authors:** Thanh G. Phan, Anisha Haseeb, Richard Beare, Velandai Srikanth, Amanda G. Thrift, Henry Ma

**Affiliations:** ^1^Department of Neurology, Monash Health, Melbourne, VIC, Australia; ^2^Stroke and Aging Research Group, Medicine, School of Clinical Sciences, Monash University, Melbourne, VIC, Australia; ^3^Department of Medicine, Frankston Hospital, Peninsula Health, Melbourne, VIC, Australia; ^4^Central Clinical School, Monash University, Melbourne, VIC, Australia; ^5^Developmental Imaging, Murdoch Children's Research Institute, Melbourne, VIC, Australia

**Keywords:** stroke, googling, map, lifetime risk, global, supplementary

## Abstract

**Objective:** We aimed to utilize the data on lifetime risk of stroke, from Global Burden of Disease (GBD) 2016, in combination with open data platforms to create an interactive map for use by clinicians and members of the public. Further, we explore the relationship between life expectancy and lifetime risk of stroke.

**Design:** Enhancing visual display of large volume of data.

**Setting:** Worldwide estimates of the lifetime risk of stroke obtained from the GBD 2016.

**Participants:** None.

**Intervention:** None.

**Methods:** Data were extracted from a portable document format (pdf) copy of the GBD article on the lifetime risk of stroke and exported into the R programming environment (version 3.4.4). These data were merged with (i) the world map boundary, (ii) open data platforms from the World Bank (life expectancy and income), and (ii) open data from the United Nation Population Prospects 2017. Further we plotted the relationship between the adjusted lifetime risk of stroke and life expectancy.

**Outcomes:** The map of the global burden of stroke shows a higher lifetime risk of stroke among high-income countries than in low-income countries (https://gntem3.shinyapps.io/strokeglobal/). The greatest risk was among upper-middle-income countries such as China and Eastern and Central European countries such as Latvia and Romania. The lifetime risk of stroke increased in countries with higher life expectancy (β = 0.48 ± 0.047, 95% confidence interval = 0.390–0.574, *R*^2^ = 0.38, *p* < 0.01).

**Conclusion:** Overall life expectancy is a major driver of the lifetime risk of stroke. The interactive map enables clinicians to search information about the lifetime risk of stroke interactively and navigate by zooming in and out, while still retaining high resolution.

## Introduction

This article was inspired by a recent publication on the global lifetime risk of stroke from the Global Burden of Disease (GBD) 2016 collaborators (1). The GBD collaborators estimated that the global lifetime risk of stroke from the age of 25 years was one in four and that the risk was twice as high for ischemic than hemorrhagic stroke. Further, the authors provided a choropleth (thematic) map illustrating the global effect of this disease, with the color on the map reflecting the lifetime stroke risk. A drawback is that the map is provided in a static image format and does not retain high resolution with zooming, nor does it easily lend itself to an interactive search. To find data for a country of interest, the reader would need to switch between the map in the main article and the 125 pages of data in the supplementary material (29.1 MB of data). The reader would struggle to easily extrapolate exact information on the lifetime risk of stroke from the map given the information is binned to a range of values. Further readers may not access the supplementary material (2, 3).

In this article, we have created a dynamic vectorized map on the web incorporating data published from the GBD 2016 global burden of stroke. Data on lifetime risk of stroke and other variables from open data sources can be obtained upon clicking the country of interest (1). Further, we bring forth in the choropleth map the remaining body of work by the GBD 2016 collaborators on ischemic and hemorrhagic stroke, available in the supplementary material.

## Methods

### Data From GBD 2016

Data in the *New England Journal of Medicine* article on lifetime risk of stroke were extracted from portable document format (pdf) copy of the article using *pdftables* library in the R programming environment. The data were exported as Microsoft Excel files. These data were imported into R for merging with the World Bank data described below (version 3.4.4) (1). This task was performed using *stringr* library to ensure that the names of the countries matched between the different datasets. The method for estimating the lifetime risk of stroke is detailed by the GBD 2016 investigators in the *New England Journal of Medicine* article (1). In brief, the GBD 2016 investigators (1) based their estimate of the lifetime risk of stroke on stroke incidence, cause-specific mortality, and all-cause mortality at a global level (defined by 7 GBD super regions and 21 GBD regions) and at a national level (195 countries) (4, 5).

### Open Data

Data on country income and life expectancy were obtained from The World Bank and comprise an Atlas Method conversion factor for classifying economy (6, 7).

### Map

The interactive thematic map was created using *leaflet* library in R (8). We added several functions to enhance user experience. The map of the world was color coded to reflect the estimated lifetime risk of stroke. Within *leaflet*, a popup function is added to enable the reader to query the data by clicking on to the country of interest.

The *Shiny* library in R is used to build a web-based app (application) with files uploaded onto the internet. We wrote a function to take the argument “country of interest” so that readers can search within the map data for that country. A “fuzzy text matching” approach is used so that even if the user mistyped “Australi” instead of “Australia,” the search path would lead the user to the correct country. The tile map we had chosen was “Open Street Map.”

### Plots

We explored the relationship between the lifetime risk of stroke and life expectancy, National income, and location of countries by latitude and continents. The plots were created within R using the *ggplot2* (9) library (Grammars of Graphics style) and expressed as a *plotly* (10) object for user interaction. This way, the plots of lifetime risk by latitude, continent, income, and life expectancy are all interactive. The reader can hover over the data point to obtain similar information on each country.

### Pseudocode


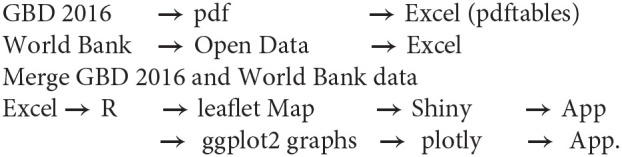


### Statistical Analysis

Lifetime risk of stroke was regressed against and life expectancy of each country. This step was repeated for each country income group. The (11) test of decreasing trend was performed for the countries, ordered by income groups (high to upper middle, lower middle, and low income).

## Results

**Figure 1:**
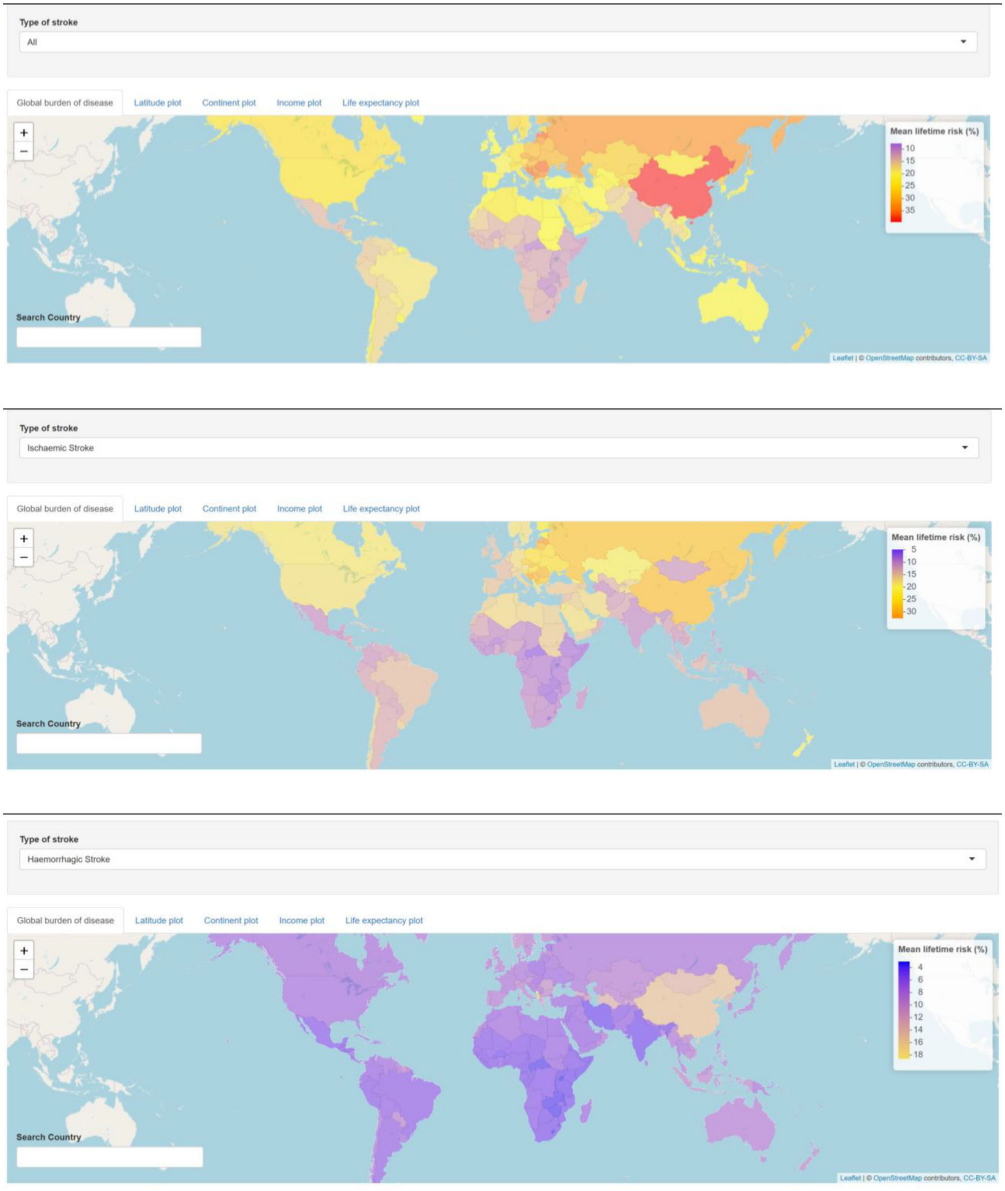
Global reach of stroke. Choropleth (thematic) map illustrating lifetime risk of all stroke (top), ischemic stroke (middle), and hemorrhagic stroke (bottom). Red illustrates a high lifetime risk of stroke, and blue, a low lifetime risk. The user can choose any of these maps by clicking the drop-down icon. The map can be accessed at https: //gntem3.shinyapps.io/strokeglobal/.

**Figure 2:**
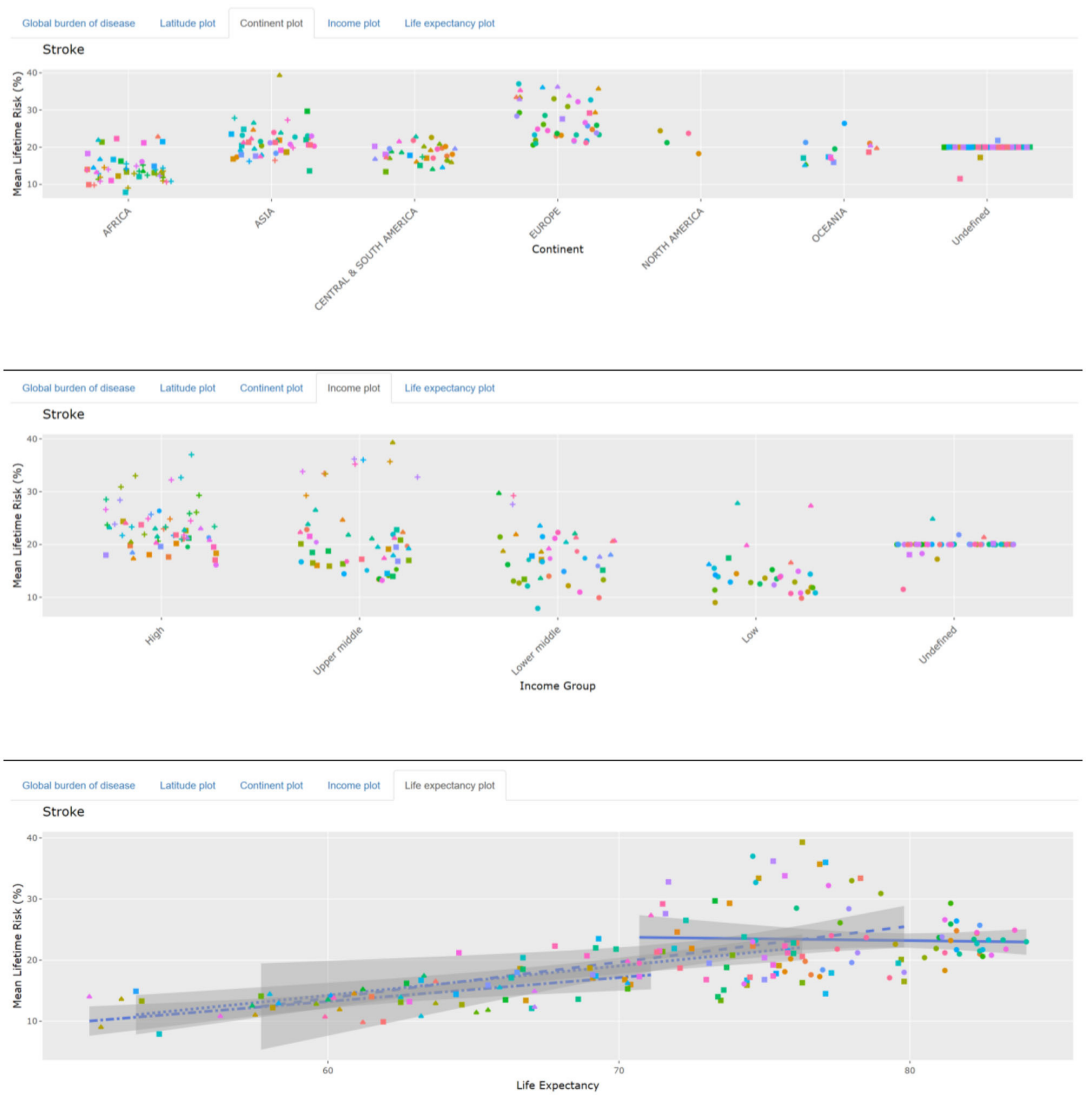
Lifetime risk of stroke vs. continent, economy, and life expectancy. On the web, the plots of lifetime risk by latitude, continent, income, and life expectancy are interactive, and the reader can hover over the data point to obtain similar information on each country. The top figure shows relationship between lifetime risk of stroke and countries grouped by continent, with risk highest in Europe and lowest in Africa. The middle figure shows decreasing trend in lifetime risk of stroke from high- to low-income countries. The lower figure shows that the relationship between lifetime stroke risk and life expectancy was β = 0.48 ± 0.047; 95% CI = 0.390–0.574; *R*^2^ = 0.380; *p* < 0.01. Subgroup analysis indicated that this relationship was strong for low-income countries (*p* < 0.01), and the relationship flattened out for high-income countries (*p* = 0.8).

The interactive map of global burden of stroke shows high estimated lifetime risk among countries with high income and Eastern European countries with upper middle income (https://gntem3.shinyapps.io/strokeglobal/). The lowest lifetime risk of all stroke is observed in African countries with low income (Figure 1). The risk of all stroke was greatest in countries with a higher life expectancy [β = 0.48 ± 0.047, 95% confidence interval (CI) = 0.390–0.574, *R*^2^ = 0.380, *p* < 0.01]; for every year increase in life expectancy, the lifetime stroke risk increases by ~0.48% (Figure 2). Outliers in this figure include upper-middle-income countries, such as China, and Eastern and Central European countries, such as Latvia and Romania. The identity of these countries can be easily accessed by hovering on the plot.

### Continents

The mean in lifetime stroke risk is highest in Europe (27.7 ± 5.1), North America (21.9 ± 2.8), Asia (21.5 ± 4.2), Oceania (18.7 ± 3.0), Central and South America (18.2 ± 2.3), and Africa (14.2 ± 3.5).

### World Bank Income

The relationship between lifetime stroke risk and life expectancy for low-income countries was β = 0.392 ± 0.108, 95% CI = 0.357–0.426, *R*^2^ = 0.322, *p* < 0.01; for lower-middle-income countries, it was β = 0.482 ± 0.102, 95% CI = 0.281–0.683, *R*^2^ = 0.362, *p* < 0.01; for upper-middle-income countries, it was β = 0.590 ± 0.216, 95% CI = 0.216–1.000, *R*^2^ = 0.140, *p* = 0.01. The relationship between lifetime stroke risk and life expectancy was not significant for high-income country (β = −0.0568 ± 0.194, 95% CI = −0.438–0.325, *R*^2^ = 0.085, *p* = 0.8).

### Hemorrhagic Stroke

The estimated lifetime risk of ischemic and hemorrhagic stroke can be viewed by clicking on the tab “Type of Stroke.” China has the greatest lifetime risk of both ischemic (28.5%) and hemorrhagic (15.5%) stroke (Figure 1). With the exception of China and Mongolia, the lifetime risk of hemorrhagic stroke in Asia is similar to Oceania or Europe and ~8%. The pattern of high lifetime ischemic stroke risk in Eastern and Central Europe did not exist for hemorrhagic stroke. In Europe, the only countries with a high lifetime risk of hemorrhagic stroke were Montenegro (18.6%) and Albania (17.1%).

The map functions on web engines such as Microsoft Edge, Google Chrome, and Mozilla. An internet connection is required to use the map.

## Discussion

We have created an interactive choropleth map of the global lifetime risk of all stroke in 2016 in an accessible format that can be viewed by anyone with internet access. Lifetime risk of stroke denotes the likelihood of suffering from a stroke in one's lifetime. Several additional features were added to explain the observation of high lifetime risk of stroke among high-income countries and selected countries in Eastern Europe with upper middle income. It is proposed that the readers view this article as a visual display of the large body of work by the GBD 2016 collaborators and refer to the original articles regarding in-depth discussion of the method and results for generating statistics (1).

The collaboration between data and/or citizen scientists and health care researchers was made possible by the availability of data from GBD 2016 investigators (1) and open data from the United Nations (7) and World Bank (6, 12). The key aspect of this article is the conversion from pdf to Microsoft Excel and uploading of data onto the web. This aspect enables seamless access to the tabular results from GBD 2016 investigators (1). The *pdftables* library in R is not the only package that can perform this task. Other available libraries within R for extracting such data include *pdftools* and *tabulizer*. The *Shiny* library serves as a framework within R environment for creating a web-based app and without the need to learn languages such as HTML (hypertext markup language) or CSS (cascading style sheets). The approach used here is one way of bringing the vast data hidden in supplementary material out into the open (2, 3). We were fortunate to be given permission by GBD 2016 investigators to show their results in map form. While it would help readers to have access to the full data, these data are already publicly available in the original manuscript. Here, we provide a different approach to visualize the body of work.

Data on life expectancy help to explain the relationship between a low lifetime risk of stroke and low-income countries. The life expectancy in low-income countries is <60 years, some 20 years less than that in high-income countries (~80 years). As the risk of stroke increases with age, stroke will be more common in countries with a greater life expectancy than in countries where people die at younger ages. For example, in Japan, the high lifetime risk of stroke is at least partially attributable to a high life expectancy (84 years). In contrast, in Somalia, the relatively low lifetime risk of stroke (10.8%) is likely largely attributable as people do not live to ages where stroke most commonly occurs, as life expectancy is only 56.3 years. Indeed, the low risk observed in the low-income countries (such as Sub-Saharan Africa including Somalia) might be explained by the high risk of death from any cause other than stroke at younger ages.

There are some worrying signs of very high lifetime risk of stroke among selected high- and upper-middle-income countries in Eastern and Central Europe. In this region, countries such as Bosnia, Latvia, and Romania have a lifetime risk of stroke >35%, whereas the risk is <30% in Poland, Moldova, and Ukraine (Figures 1, 2) (6). Overall, these risks are much higher than those in Western and Southern European countries. The exception in Eastern Europe is Estonia, which has similar lifetime stroke risk as Western Europe. In an earlier study from GBD 2013 investigators, it was proposed that 74.2% of stroke burden was due to behavioral factors such as smoking, poor diet, and low physical activity (13). It is not clear if additional factors are at play in these countries such as sociopolitics and organization of health services (14, 15). Elsewhere there has been an 11.8% decline in the all-cause burden of disease attributable to smoking in high-income countries (15).

The pattern described above is driven by the lifetime risk of ischemic stroke. Globally, it appears that the lifetime risk of hemorrhagic stroke is similar between countries, with exceptions being China, Mongolia, Albania, Montenegro, and Turkmenistan. This pattern is somewhat different from the previously reported high risk of intracerebral hemorrhage in Asians (16). One possible explanation is that the high lifetime risk of hemorrhagic stroke may be limited to Chinese and Mongolians, the former being the world's most populous nation (15). The GBD 2016 had combined subarachnoid hemorrhage and intracerebral hemorrhage into hemorrhagic stroke data. However, this should not significantly affect the quality of the data as subarachnoid hemorrhage forms a very minor subset of all stroke (14).

### Limitations

The GBD 2016 collaborators provided lifetime risk on most country but noted that data were not available in some countries (1). The GBD 2016 collaborators had acknowledged this limitation in their article and had discussed the assignment of values of neighboring countries and data on country-level risk exposure (1). It should be noted that the World Bank has changed the status of some high-income countries over time, and this change has affected countries such as Russia and Venezuela; these countries are now listed as upper-middle-income countries (6). One advantage of this dynamic map is that it can easily be updated should the GBD 2016 collaborators wish to make changes.

## Conclusion

An interactive thematic map extends the value of the excellent work by the GBD 2016 collaborators on the lifetime risk of stroke.

## Article Summary

### Strength

(1) Data are based on the adjusted lifetime risk of stroke that come from GBD 2016 collaborators.

(2) The interactive web display is available at https://gntem3.shinyapps.io/strokeglobal/.

(3) The plots of lifetime risk by latitude, continent, income, and life expectancy are interactive.

## Permission

On 20th April 2020, the GBD team provided written permission to use the data. They noted that “any data made available for download on IHME Websites can be used, shared, modified, or built upon by non-commercial users via the Open Data Commons Attribution License. Our full Terms and Conditions can be found here.”

## Data Availability Statement

Publicly available datasets were analyzed in this study. This data can be found here (15): the GBD 2016 investigators indicated that data can be found on the IHME website.

## Ethics Statement

Ethical review and approval was not required for the study on human participants in accordance with the local legislation and institutional requirements. Written informed consent for participation was not required for this study in accordance with the national legislation and the institutional requirements.

## Author Contributions

TP: design. AH, TP, and RB: data extraction, statistical analysis, and mapping. TP, AH, RB, HM, VS, and AT: manuscript writing. All authors contributed to the article and approved the submitted version.

## Conflict of Interest

The authors declare that the research was conducted in the absence of any commercial or financial relationships that could be construed as a potential conflict of interest.
